# Bovine Adenoviral Vector-Based Platform for Vaccine Development

**DOI:** 10.3390/vaccines13050494

**Published:** 2025-05-03

**Authors:** Ekramy E. Sayedahmed, Vivek Gairola, Muralimanohara S. T. Murala, Suresh K. Mittal

**Affiliations:** Department of Comparative Pathobiology and Purdue Institute of Inflammation, Immunology and Infectious Disease, College of Veterinary Medicine, Purdue University, 625 Harrison St., West Lafayette, IN 47907-2027, USA; esayedah@purdue.edu (E.E.S.); vgairola@purdue.edu (V.G.); mmurala@purdue.edu (M.S.T.M.)

**Keywords:** bovine adenovirus, pathogenesis, virion structure, genome organization, bovine adenoviral vector-based platform, nonhuman adenoviral vector system

## Abstract

Adenoviral (AdV) vector-based vaccines employing the human AdV (HAdV) and chimpanzee AdV (ChAdV) vector platforms played a crucial role in combating the COVID-19 pandemic. However, the widespread use of these platforms, the prevalence of various HAdV types, and the resulting preexisting immunity have significantly impacted the vaccines utilizing these vector platforms. Considering these challenges, the bovine AdV type 3 (BAdV-3) vector system has emerged as a versatile and innovative platform for developing next-generation vaccines against infectious diseases. Inherent attributes like a high transduction efficiency, large transgene insertion capacity, broad tissue tropism, and robust induction of innate immunity add significant value to the BAdV vector platform for vaccine design. BAdV-3 vectors effectively elude HAdV-specific preexisting humoral and cellular immune responses. Additionally, BAdV-3 is low in pathogenicity for its host and is anticipated to be safe as a vaccine platform. This systematic review provides an overview of the development of BAdV-3 as a vaccine delivery platform and its application in designing vaccines for infectious agents of human and veterinary importance.

## 1. Introduction

**Figure 1:**
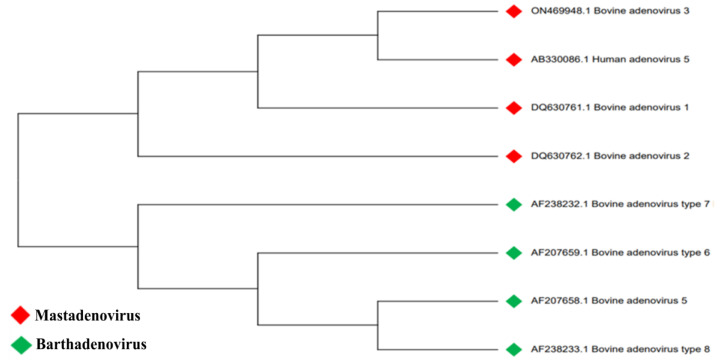
Phylogenetic tree of BAdVs based on amino acid sequences of the hexon protein. The phylogenetic tree shows the grouping of *Mastadenovirus* members along with HAdV-5 in one cluster and the *Barthadenovirus* members in another cluster. The amino acid sequences of hexon protein were retrieved from the NCBI Genome database.

Adenoviruses (AdVs) are non-enveloped icosahedral viruses with a 25–48 kilobase pair (kbp), non-segmented, double-stranded linear DNA genome, and belong to the *Adenoviridae* family [1]. The *Adenoviridae* family has six genera: *Mastadenovirus*, *Aviadenovirus*, *Ichtadenovirus*, *Siadenovirus*, *Testadenovirus*, and *Barthadenovirus*, infecting a wide range of hosts, including humans, nonhuman primates, cattle, pigs, dogs, cats, rodents, birds, fish, and reptiles [2,3]. Bovine AdVs (BAdVs) commonly infect cattle’s respiratory and/or digestive tracts, leading to either asymptomatic or mild disease. Clinical disease is usually associated with stressful conditions, including shipping and cold weather [2]. Based on the virus neutralization assay, ten types of BAdVs are grouped into two genera: BAdV-1, BAdV-2, BAdV-3, BAdV-9, and BAdV-10 belong to the *Mastadenovirus* genus, while BAdV-4, BAdV-5, BAdV-6, BAdV-7, and BAdV-8 are in the *Barthadenovirus* genus [3] (Figure 1). A recent BAdV isolate from cattle in Europe was proposed as BAdV-11 [4].

There has been a growing interest in AdV vector-based platforms for developing vaccines against infectious diseases in recent years. AdV vector-based vaccines were pivotal in controlling the COVID-19 pandemic, with more than 3.8 billion doses administered globally. These vaccines were primarily based on human AdV (HAdV) and chimpanzee AdV (ChAdV) vector systems. However, preexisting vector immunity against ~100 HAdV genotypes prevalent in the human population severely limits the use of many HAdV-based vectors [5]. To tackle this bottleneck, rare HAdV types and several nonhuman AdVs, *viz*., BAdV, porcine AdV (PAdV), simian AdV (SAdV), canine AdV (CAdV), ovine AdV (OAdV), and avian AdV, have been utilized to develop vectors [6]. Among these, the BAdV-3 vector stands out due to its unique features. BAdV-3 demonstrates no cross-neutralization with HAdV-specific antibodies and limited cross-reactivity to HAdV-specific T-cell responses, thereby overcoming the challenges posed by preexisting AdV vector immunity [7,8]. BAdV-3 vectors are safe, have prolonged persistence in vivo, lead to robust activation of innate immunity, and are suited for intranasal delivery due to the usage of α(2,3)- and α(2,6)-linked sialic acid receptors [9,10,11,12].

This systematic review provides a comprehensive overview of the BAdVs, including their pathogenic potential, virion structure, genome organization, and replication strategy. The development of the BAdV-3 vector platform, along with its salient features and application in vaccine development, has also been discussed in detail. Multiple databases, including PubMed, Google Scholar, and ScienceDirect, were accessed using the following keywords: bovine adenovirus, bovine adenovirus-associated diseases, bovine adenoviral vector-based platform, bovine adenovirus genome organization, and bovine adenoviral vector-based vaccines. All the articles relevant to BAdV-associated diseases, pathogenesis, virion structure, genome organization and replication, vector platform development, and BAdV vector-based vaccines were selected.

## 2. BAdV-Associated Diseases 

BAdVs have a worldwide distribution, and several serological surveys have demonstrated their prevalence in the bovine population [13,14,15]. Despite being highly prevalent, they are seldom associated with overt diseases [16]. BAdVs predominantly cause inapparent to mild infection in cattle, as evidenced by the identification of initial isolates in healthy animals [17,18,19].

Calves aged between 3 weeks and 4 months are highly prone to infection with BAdVs. Maternal antibodies in the colostrum are pivotal in providing type-specific protection against BAdV infection in young calves [20]; therefore, calves deprived of colostrum and post-weaners have higher susceptibility. Additionally, exposure to heterologous BAdV types in the presence of stressors like improper nutrition, shipping, overcrowding, and temperature extremes will further predispose the calves to infection [20,21]. Viral transmission is mainly through the oral or nasal route [20]. The incubation period relies upon the route of inoculation and generally ranges from 7 to 10 days [22]. Clinical signs include pyrexia (39.5–40.5 °C) and anorexia, followed by respiratory and gastrointestinal manifestations. Respiratory signs usually occur first, with serous to mucoid nasal and ocular discharge, dyspnea, and coughing [23]. Gastrointestinal signs range from salivation and mild diarrhea to severe dysentery due to vascular damage caused by other viruses [16,23]. Conjunctivitis, keratoconjunctivitis, and polyarthritis may also occur in some cases [24,25]. Infection results in diminished weight gain owing to malabsorption. During acute infection, the virus is shed in feces, urine, nasal discharge, and conjunctival secretions [20,23]. However, in animals with latent kidney infection, virus secretion in urine may continue for up to 10 weeks or more [20]. The nature and severity of clinical signs depend upon the type of BAdV involved. BAdV-1, -2, -4, and -8 are usually associated with respiratory signs and pneumoenteritis [26,27,28].

BAdV-3 infection usually leads to pneumoenteritis [23]; however, severe gastrointestinal involvement leads to multifocal hemorrhagic and necrotic lesions in the rumen, omasum, abomasum, small intestine, and colon [16]. BAdV-3 has also been linked to cases of conjunctivitis and keratoconjunctivitis [25]. Upon experimental intranasal (i.n.) inoculation with BAdV-3, two-year-old heifers with variable levels of BAdV-3 neutralizing antibody titer (ranging from 120 to 1080) showed no clinical signs for ten days post-inoculation. However, there was a significant increase in BAdV-3-specific serum IgG, IgG_1_, or IgG_2_ ELISA titers and virus-neutralizing antibody titers following BAdV-3 infection [29]. Following i.n. infection of cotton rats (*Sigmodon hispidus*) with BAdV-3, virus replication was observed in the lung and trachea, characterized by pneumocyte type II hyperplasia in the alveoli, peribronchiolar and perivascular lymphocytic infiltration, necrosis and hyperplasia of bronchiolar epithelium, and eosinophilic intranuclear inclusions peaking at day 3, indicating that the cotton rat is a suitable animal model for evaluating the efficacy of replication-competent or replication-defective BAdV vectors [30].

The primary manifestation of BAdV-5 infection in calves is polyarthritis or weak calf syndrome [24]. BAdV-6 infection has been associated with severe dysentery [14]. BAdV-7 infection results in respiratory signs, diarrhea, polyarthritis, or weak calf syndrome [13,31,32]. BAdV-7 has also been reported in cervids. In a seroprevalence study involving sika deer (*Cervus nippon yesoensis*) in Japan, 54% of farmed deer and 21% of the wild deer populations under study had antibodies against BAdV-7. However, the role of BAdV-7 in the occurrence of clinical disease is unclear [33]. BAdV-10 is considered highly pathogenic and has been associated with fatal hemorrhagic enterocolitis in calves due to severe vascular damage [34,35].

AdVs have also been implicated in inducing tumor formation in laboratory animals, but not in their natural host. So far, only two BAdVs, i.e., BAdV-3 (strain WBR-1) and BAdV-8 (strain 6833), have been reported to induce tumor formation in Syrian hamsters following inoculation by multiple routes [36,37].

## 3. Virion Structure

**Figure 2:**
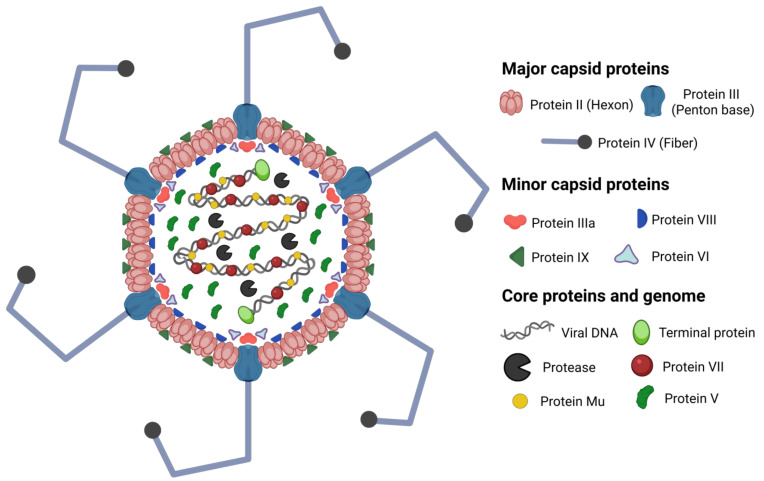
Illustration of the BAdV-3 virion structure. The BAdV-3 fiber is long and bent. The capsid proteins are hexon, penton base, IIIa, pVI, pVIII, and pIX. The internal virion proteins are pV, pVII, pX (Mu), protease, and terminal protein. The distribution of the internal protein in the virion is hypothetical. The terminal protein is attached to the 5ʹ end of the viral genome at both ends.

The AdV capsid is composed of 240 capsomers made of hexon protein. The capsid has 12 vertices containing the penton base protein and a protruding fiber. The fiber consists of a fiber shaft and a fiber knob. BAdVs have 13 different structural polypeptides, named pII (hexon), pIII (penton base), pIIIa, pIVa2, pIV (fiber), pV (only in *Mastadenovirus*), pVI, pVII, pVIII, pIX (only in *Mastadenovirus*), pX (Mu), protease, and terminal protein [38] (Figure 2). BAdV-3 fiber is exceptionally long compared to several other AdVs due to the increased number of repeating motifs in the shaft region [39]. Electron microscopic examination of purified BAdV fiber revealed that it is bent in several places, matching irregular shaft repeats [40]. The fiber’s bending was hypothesized to be critical for the interaction of the penton base with the secondary receptors. The crystal structure of the BAdV-4 fiber head domain at 1.2 Å resolution was determined, and it shows similarity with the OAdV-7 fiber head [41].

## 4. Genome Organization

**Figure 3:**
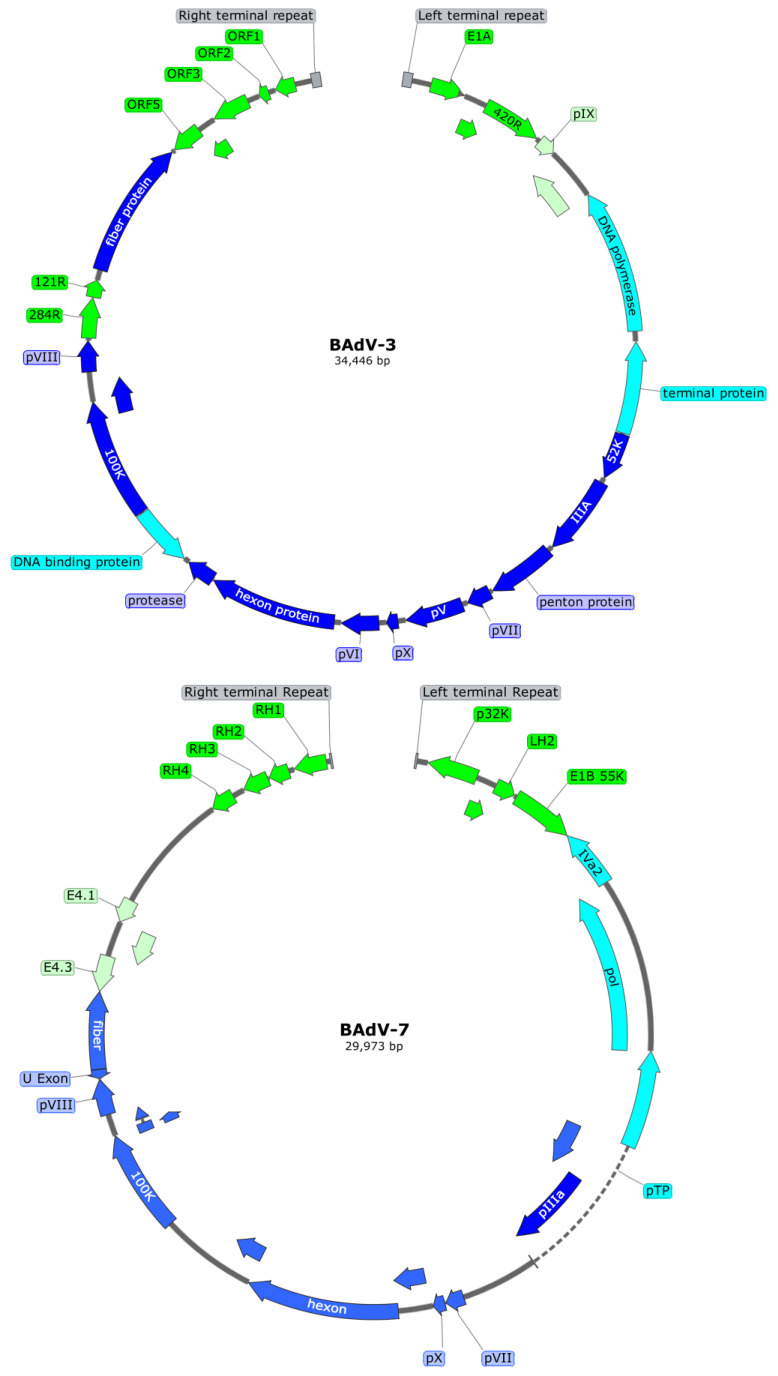
The genome organization of BAdV-3, belonging to *Mastadenovirus* genus, and BAdV-7, belonging to *Barthadenovirus* genus. ORF, open-reading frame; pTP, pre-terminal protein.

The double-stranded, non-segmented linear DNA genomes of the BAdVs have inverted terminal repeats (ITRs) on both ends. Each 5ʹ end of the genome is linked to a terminal protein. BAdV-3 is the most studied BAdV, and its genome is 34,446 bp long with 195 bp ITRs [38]. The right and left ends of the genome after ITRs encode for the nonstructural proteins and are highly variable between different BAdVs. The central part of the genome mainly encodes the virus structural proteins and is relatively consistent across the *Adenoviridae* family members [1,2]. The genome of the *Mastadenovirus* BAdV-3 has four early (E) regions: E1 to E4. The E1 and E4 regions are located at the left and right ends of the genome, respectively, and encode proteins responsible for the modulation of the host cell transcriptional machinery to facilitate AdV replication. The E3 region is responsible for the modulation of the host immune system to avoid detection by the host’s immune system and gives the virus a chance to replicate before the lysis of the infected cells. The E2 region encodes the DNA polymerase, DNA-binding protein, and the terminal protein required for the virus DNA replication. The central region of the AdV genome has five late gene regions encoding most of the AdV structural proteins essential for virus assembly [2,38] (Figure 3).

## 5. Receptors and Cell Entry

AdV attachment to the permissive host cell involves two sequential steps, initial high-affinity binding of the fiber knob with the primary receptor and subsequent interaction of the penton base with secondary receptor, leading to internalization by clathrin-mediated endocytosis [42]. For most HAdVs, coxsackievirus-Ad receptor (CAR) acts as the primary receptor, while integrins (α_v_β_3_ and α_v_β_5_) serves as the secondary receptor [42,43]. However, experimental evidence based on amino acid sequences of the BAdV-3 fiber and knob, CAR and integrin receptor blocking assays, and knob-mediated competition assays suggest that BAdV-3 uses a receptor distinct from CAR and integrins [44].

The fiber and knob domain amino acid sequence similarities between BAdV-3 and HAdV-5 were 29.3% and 25.3%, respectively. The BAdV-3 fiber’s shaft region (976 residues, 46.5 repeats in shaft) was comparatively longer than the HAdV-5 fiber (581 residues, 22 repeats in shaft). Additionally, the BAdV-3 knob lacked residues essential for binding with CAR and the penton base lacked integrin binding motifs, i.e., RGD (arginine–glycine–aspartic acid) or LDV (leucine–aspartic acid–valine) [44]. BAdVs instead possess an MDV (methionine–aspartic acid–valine) motif, but its role in integrin binding is unclear [38].

There was no inhibition of BAdV-3-GFP (BAdV-3 expressing the green fluorescent protein) transduction of Madin Darby bovine kidney (MDBK) cells upon blocking the CAR and α_v_β_3_ integrin with respective monoclonal antibodies. Preincubation of MDBK cells with a recombinant BAdV-3 fiber knob domain inhibited the transduction of BAdV-3-GFP, but no inhibition was seen for HAdV-5-GFP. Similarly, MDBK cells preincubated with the recombinant HAdV-5 fiber knob domain inhibited HAdV-5-GFP, but not BAdV-3-GFP. Taken together, these results indicate that BAdV-3 employs an entry receptor distinct from that of HAdV-5 [44].

The potential role of sialic acid was explored in the search for the entry receptor used by BAdV-3. Neuraminidase treatment (0.1 to 10 mU/mL) of MDBK cells to remove sialic acid residues from the cell surface lowered BAdV-3-GFP transduction by up to 93%. Further, blocking sialic acid residues by pretreating MDBK cells with wheat germ agglutinin lectin (WGA) (≥0.05 mg/mL) diminished BAdV-3-GFP transduction by approximately 97% [9]. Additional experiments involving linkage-specific lectins, i.e., *Maackia amurensis* agglutinin (MAA) [blocks α(2,3)-linked sialic acid] and *Sambucus nigra* agglutinin (SNA) [blocks α(2,6)-linked sialic acid], were conducted to ascertain the exact nature of the sialic acid receptor. Reductions of 59% and 90% were observed in the BAdV-3-GFP transduction of MDBK cells upon preincubation with SNA (0.5 mg/mL) or MAA (0.5 mg/mL), respectively. Hence, BAdV-3 employs both α(2,3)- and α(2,6)-linked sialic acid residues as entry receptors with greater affinity for α(2,3)-linked sialic acid. It was further determined that BAdV-3-GFP transduction of MDBK cells is sensitive to pretreatment with sodium periodate (NaIO_4_), which selectively oxidizes and destroys carbohydrates, indicating that the BAdV-3 receptor is a sialic acid-bearing carbohydrate. The location of sialic acid was traced to cell surface glycoproteins (97 kDa and 34 kDa), as trypsin treatment lowered the BAdV-3-GFP transduction. Hence, BAdV-3 uses α(2,3)- and α(2,6)-linked sialic acid residues on the cell surface glycoproteins as primary entry receptors [9].

## 6. Virus Replication

The replication of BAdVs has not been studied in detail, and the reports on the exact replication mechanism are scarce. However, most proteins involved in genome replication are conserved among AdVs with shared functional homologies. Hence, the following description provides a general overview of AdV replication and incorporates certain characteristic features of BAdV-3 replication.

Following endosome-mediated virus internalization, the mechanical force generated by biophysical interactions destabilizes the capsid structure. The fiber and penton base are shed, resulting in the loosening of the capsid vertices [45]. This releases protein VI (membrane lytic protein) from the partially disassembled capsid into the endosome. Protein VI-mediated destruction of the endosomal membrane, accompanied by a concomitant decrease in the endosomal pH, leads to the escape of partially uncoated virions into the cytosol [46]. These AdV virions bind to dynein microtubule motor proteins mediated through hexon and protein VI, and are transported to the nuclear pore complex (NPC) [46,47]. These virions are docked at the NPC by the interaction of hexon with Nup214 (nuclear pore filament protein) [48]. They further interact with kinesin-1 and histone H1. The mechanical pull resulting from the movement of kinesin-1, accompanied by binding to H1, culminates in the complete disruption of the capsid. The AdV genome and core proteins are then imported into the nucleus [49,50].

In the susceptible host, AdV generally infects differentiated cells that rarely divide. The AdV genome in the nucleus employs RNA polymerase II to transcribe E1A and E1B genes, to make these cells conducive for virus replication. BAdV-3 E1A proteins 211R, 115R, and 100R induce the expression of E2, E3, and E4 viral genes and some cellular genes [51]. The binding of E1A proteins to retinoblastoma (Rb) tumor suppressor protein releases cellular transcription factor E2F, thereby inducing the host cell to enter the DNA synthesis/S phase [52]. However, in doing so, they indirectly trigger apoptosis by activating the tumor suppressor p53 protein [53]. E1B proteins E1B^small^ (157R) and E1B^large^ (420R) counter this pro-apoptotic potential of the E1A proteins by suppressing p53-induced genes and ubiquitin-mediated proteolysis of p53 [52,54].

Once the cell enters the S phase, the collaborative effort of E2-encoded proteins, i.e., DNA polymerase (Pol), pre-terminal protein (pTP), and DNA-binding protein (DBP) leads to the replication of viral genomic DNA [2]. Host transcription factors neurofibromin 1 (NF1) and octamer-binding transcription factor 1 (Oct-1; NFIII) have been reported to augment replication. They mobilize Pol, pTP, and DBP to the origin of viral genome replication (Ori) to form a preinitiation complex [55]. The AdV Pol is a family B polymerase that requires a protein primer and has 3ʹ to 5ʹ exonuclease (proofreading) activity [56]. pTP remains covalently linked to the 5ʹ end of viral DNA and functions as a protein primer, initiating replication at either end of the AdV genome [57]. The viral Pol catalyzes the formation of a phosphodiester bond between the β-hydroxyl group of the serine residue of pTP and deoxycytidine monophosphate (dCMP). The viral Pol uses the free 3ʹ-OH group on dCMP as a primer and progresses along the linear AdV genome for chain elongation [58]. This results in progeny duplex DNA and a displaced parental single-stranded (ss) DNA strand. DBP is associated with the displaced ssDNA (1 monomer/10–15 nucleotides), protecting it from nucleases and regulating its self-annealing along ITRs [59,60]. This forms a panhandle structure with Ori, initiating another round of DNA replication [61]. AdV genome replication and subsequent transcription occur in specialized nuclear compartments known as viral replication compartments (VRCs) [60].

The viral DNA replication is followed by transcription of the intermediate gene unit and synthesis of IVa2 and pIX proteins. IVa2 functions as a transcriptional activator for the AdV major late promoter (MLP) and enhances the expression of late genes from the AdV major late transcription unit (MLTU) [62]. Multiple transcripts are generated from MLTU by alternative splicing. The resultant proteins include the major capsid proteins (III/penton base, IV/hexon, and fiber), minor capsid proteins (IIIa, pVI, pVIII, and pIX), viral core proteins (pV, pVII, µ, and protease), and nonstructural proteins (22K, 33K, 52K, and 100K) [62]. Additionally, all MLTU transcripts share a common 5ʹ non-coding sequence known as the tripartite leader (TPL), which efficiently transports them to the cytoplasm and enhances their translation [38]. The translated late proteins possess a nuclear localization signal (NLS), which binds to the nuclear transport receptors, transportins, or importin α/β, resulting in their nuclear transport via NPC [63].

AdV assembly commences in the nucleus, where chaperons or scaffolding proteins 33K, 52K, and 100K regulate the assembly of structural and core proteins to form empty capsids [64,65]. Next, the packaging proteins IVa2, 22K, 33K, and 52K bind to the *cis*-acting packaging sequences positioned at the left end of the AdV genome between the left ITR and E1A region and initiate genome packaging into empty capsids [66,67]. The IVa2 protein functions as a packaging motor and ATPase, along with small terminase proteins (22K and 33K), to package the AdV genome into the empty capsid via a putative portal formed by the E4–34K protein [67,68]. For final maturation, viral protease cleaves precursor structural proteins pIIIa, pTP, pVI, pVI, pVIII, and µ and degrades scaffolding proteins that are subsequently released from the assembled virions [69]. Mature infectious virions are released from the host cell by cell lysis.

## 7. BAdV Vectors for Gene Delivery

AdV vectors have garnered considerable attention for gene therapy and vaccine development due to their commendable safety profile, substantial cloning capacity, ease of production of high-titer stocks, and efficient transduction of several cell types. Nevertheless, the practical applications of several AdV vectors encounter challenges, such as poor targeting due to broad tissue tropism and robust immune responses against the vector backbone, thereby impeding their reusability [70]. In addition, the widespread AdV vector immunity against highly prevalent HAdVs poses another challenge [5,71]. Alternate human and nonhuman AdV vector platforms were pursued to mitigate some of the shortcomings of conventional AdV vectors. The BAdV-3 vector platform was developed to supplement other human and nonhuman AdV vectors [72].

### 7.1. Foreign Gene Cassette Insertion Sites and Methods for Generation of BAdV Vectors

To develop BAdV-3 as a gene delivery vector, the E3 and E1 regions were initially sequenced [38,39]. E3 gene products are not essential for AdV replication, whereas E1-encoded proteins E1A and E1B^large^ are vital for virus replication [54,73]. The E1B^small^ protein requirement seems cell-type-dependent [54]. The first effort to generate a replication-competent BAdV-3 vector employed homologous recombination in MDBK cells. MDBK cells were co-transfected with a shuttle plasmid containing the luciferase (Luc) gene in the E3 region and the BAdV-3 genomic plasmid to generate BAd-Luc [72]. The E3 deleted vector can potentially take up to 3 kb DNA [73]. BAd-Luc-infected MDBK or human embryonic kidney 293 (HEK293) cells showed excellent luciferase expression. Pathogenesis of BAd-Luc was compared to BAdV-3 after i.n. infection in cotton rats. Quantitative analysis revealed that the severity of histopathological lesions and intensity of immunohistochemical staining were identical in both infections [74]. Luciferase activity was observed in the lungs of the BAd-Luc-infected animal group till four days post-inoculation. In addition, luciferase- and BAdV-specific antibodies were found in BAd-Luc-inoculated cotton rats’ sera, suggesting the BAdV vector’s potential for developing virus-vectored vaccines [74].

**Figure 4:**
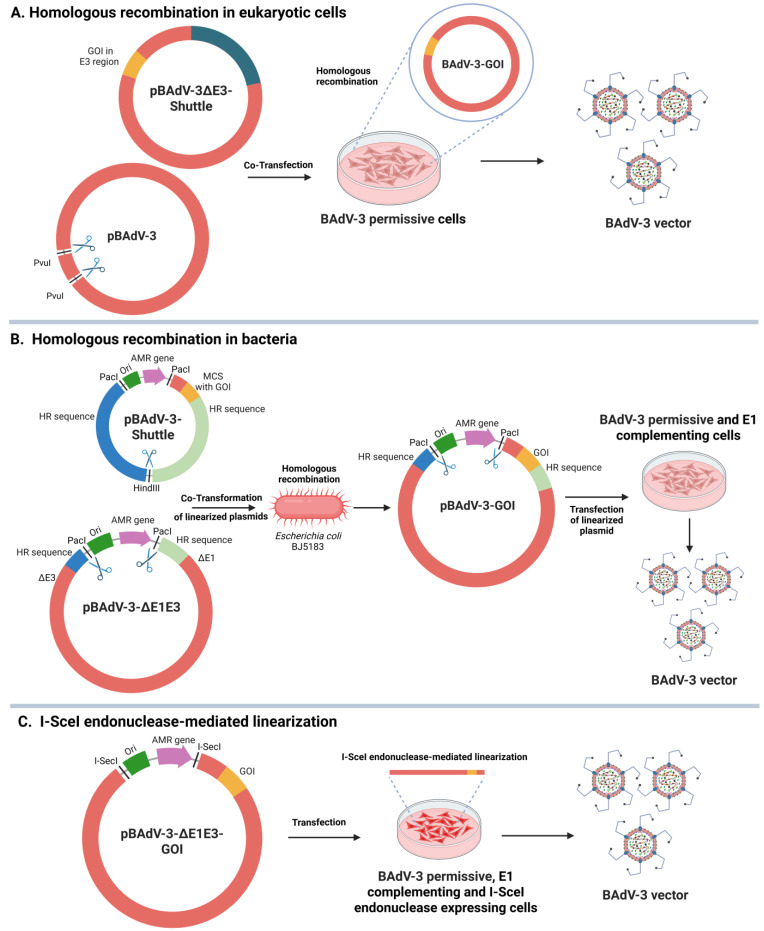
Strategies for generation of BAdV-3 vectors. (**A**) Homologous recombination in eukaryotic cells. (**B**) Homologous recombination in bacteria. (**C**) I-SceI endonuclease-mediated linearization. GOI, gene of interest; ΔE1, deletion in early region 1; ΔE3, deletion in early region 3; HR, homologous recombination; Ori, origin of replication; AMR, anti-microbial resistance; MCS, multiple cloning sites.

To further improve the efficiency of generating BAdV vectors, the homologous recombination machinery in *Escherichia coli* strain BJ5183 was utilized, followed by transfection of the restriction enzyme-digested plasmid with recombinant BAdV-3 genome into suitable E1A complementing cell lines [75,76]. Additionally, transfection of a circular plasmid with I-SceI recognition site-flanked BAdV-3 genomic DNA into cells constitutively expressing I-SceI endonuclease is a quick and efficient method for the generation of BAdV-3 recombinant vectors [77,78] (Figure 4).

Besides E1 and E3, the E4 region can also be used for developing BAdV vectors [79]. The deletion of either 1342 bp from the right end or 1501 bp from the left end of the E4 region will produce a replication-competent virus, suggesting that these areas are not essential for BAdV-3 replication. The E3–E4-deleted BAdV-3 vectors can carry ~4.5 kb, while E1A-E3–E4-deleted vectors can carry up to 5 kb of foreign DNA [80]. The maximum foreign gene insertion capacity of BAdV-3 vectors is not well-defined; however, it is expected to be at least 1 kb more than the deletion.

### 7.2. Cell Lines for Generation of BAdV Vectors

BAdV-3 grows efficiently in cell lines of bovine origin, like fetal bovine retina (FBRC), bovine fibroblast (BFB), primary fetal bovine kidney (FBK), and MDBK cells [54,81]. Cells of human origin, like HEK293, are permissive to BAdV-3 but do not support productive infection due to the block in viral genome replication and expression of late proteins. However, HEK293 cells constitutively expressing the T antigen of simian virus 40, i.e., 293T cells, overcome this block, resulting in partial productive replication of BAdV-3 [82].

However, E1 insertion vectors are replication-defective because E1 gene products are essential for virus replication. AdV recombinants containing foreign gene inserts in the E1 region can only be isolated and grown in a permissive cell line that constitutively expresses E1 proteins [54,81]. The E1A region of BAdV-3 has been reported to complement that of HAdV and vice versa, with a similar level of complementation [51,81,83].

Multiple E1-expressing cell lines have been developed for the generation of BAdV vectors. Three E1A-complementing cell lines, *viz*., MDBK-221 (MDBK cells transfected with BAdV-3 E1), FBK-34 (FBK cells transfected with BAdV-3 E1), and FBRT-HE1 (FBRT cells transfected with HAdV-5 E1), were generated [81]. These cell lines promoted replication of the E1A-deleted BAdV-3 vector. In another study, two cell lines, *viz*., 6.93.9 (MDBK cells expressing BAdV-3 E1 proteins) and VIDO R2 (FBRC cells transformed with HAdV-5 E1A/B), effectively complemented E1A deletion and supported the growth of BAdV-3 E1A deletion mutants [76]. VIDO R2 cells were more effective in generating BAdV-3 recombinants due to their higher transfection efficiency.

Another strategy involved generating bovine × human hybrid (BHH) cell lines through PEG-mediated somatic hybridization between HEK293 and MDBK cells, in order to combine their unique characteristics. This approach yielded three cell lines, *viz*., BHH3, BHH8, and BHH2C [84]. These cell lines expressed HAdV-5 E1 proteins and demonstrated a greater transfection efficiency than the parental cell lines, but only BHH3 and BHH8 cells supported the growth of BAdV-3-ΔE1A virus. The BHH3 cell line has been further modified to express BAdV-3 E1B in order to generate BHH/F5 cells, and BAdV-3 E1B along with I-SceI endonuclease to generate BHH/F5-I-SceI cells [78].

Apart from the cells of bovine origin, cotton rat lung (CRL) cells were transfected with PacI-digested plasmid containing the BAdV-3 genome, resulting in the generation of recombinant BAdV-3 [77]. Additionally, a CRL cell line stably expressing the endonuclease I-SceI, i.e., VIDO DT1, when transfected with a plasmid containing the BAdV-3 genome flanked with recognition sequences of I-SceI, led to the generation of the BAdV-3 recombinant efficiently. VIDO DT1 cells demonstrated a good transfection efficiency, with a 1.5-fold reduction in the time for rescue of recombinants and a 10-fold reduction in the amount of DNA to be transfected [77].

### 7.3. Unique Features of the BAdV-3 Vector Platform

**Figure 5:**
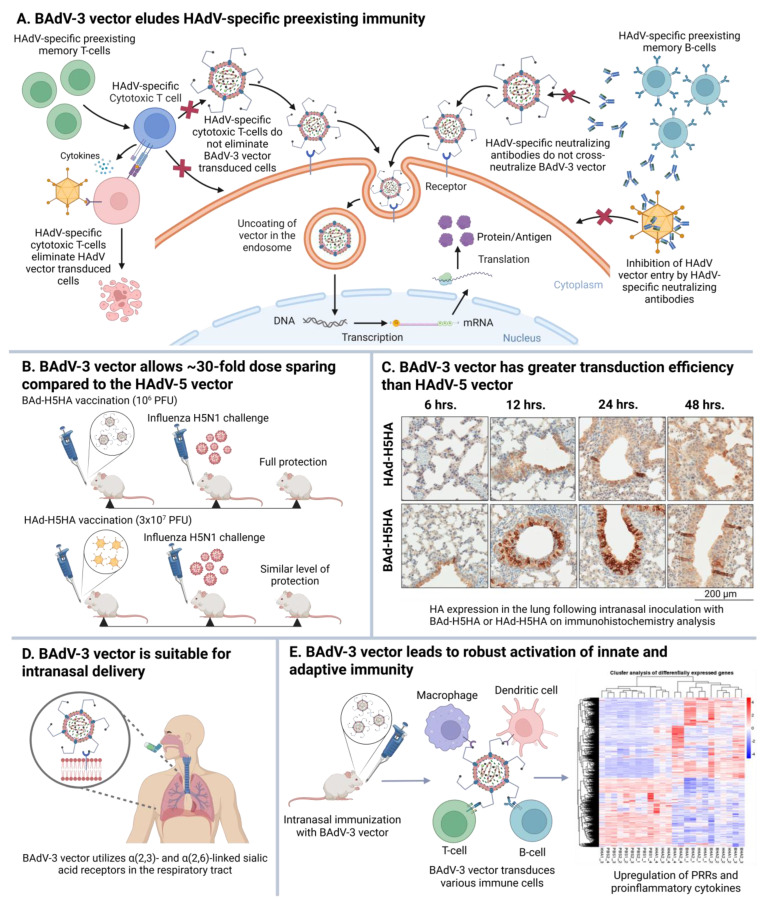
Unique features of the BAdV-3 vector platform. (**A**) BAdV-3 vector eludes HAdV-specific preexisting immunity. (**B**) BAdV-3 vector allows ~30-fold dose sparing compared to the HAdV-5 vector. (**C**) BAdV-3 vector has greater transduction efficiency than HAdV-5 vector. (**D**) BAdV-3 vector is suitable for intranasal delivery. (**E**) BAdV-3 vector leads to robust activation of innate and adaptive immunity. HA, influenza virus hemagglutinin; PFU, plaque-forming units; PRRs, pathogen recognition receptors.

The BAdV-3 vector platform exhibits several distinctive features that make it attractive (Figure 5). Antibodies raised in rabbits or mice against BAdV-3 or HAdV-5 fail to exhibit cross-virus neutralization [7]. Notably, BAdV-3-primed mice had considerably higher (*p* > 0.05) expression of the reporter gene following inoculation with HAdV-5 vector expressing β-galactosidase (HAd-LacZ) than the HAdV-5-primed group [7]. Besides the lack of cross-neutralizing antibodies, there was limited cross-reactivity among BAdV-3-, PAdV-3-, or HAdV-5-specific CD8+ and CD4+ T cells [8]. These observations highlight that the immune responses generated against one AdV do not effectively neutralize a different AdV, thereby mitigating the challenges posed by AdV preexisting immunity. In mice with exceptionally high levels of HAdV vector immunity, this did not have any impact on the level of humoral and cell-mediated immune (CMI) responses and protection efficacy of the BAdV-3 vector expressing hemagglutinin (HA) of an H5N1 influenza virus (BAd-H5HA) [85].

In contrast to CAR used by HAdVs, BAdV-3 utilizes α(2,3)- and α(2,6)-linked sialic acid receptors on the cell surface glycoproteins to enter susceptible host cells [9]. This means the BAdV-3 vector is suitable for intranasal delivery and transducing the respiratory tract’s cells, where the sialic acid receptors are abundant [10,86]. Additionally, BAdV-3 exhibits a distinct tropism compared to HAdV-5, efficiently transducing a broad range of human and nonhuman cells in culture [71]. Administration of the BAdV-3 vector via intravenous inoculation effectively transduces various organs, including the lungs, liver, spleen, heart, and kidneys. Compared to a HAdV-5 vector, the persistence of the BAdV-3 vector in a mouse model is prolonged, particularly in the heart, lungs, and kidneys [11]. In contrast to HAdV-5 and PAdV-3 vectors, BAdV-3 robustly activates Toll-like receptor 4 (TLR4) [87,88]. HAdV-5 leads to Kupffer cell depletion as it is efficiently taken up by the liver Kupffer cells due to the interaction of their negatively charged capsid with scavenger receptors. This results in the rapid clearance and lower persistence of the HAdV-5 vector. However, BAdV-3 vector administration in mice does not result in Kupffer cell depletion, as they possess a considerably less negatively charged capsid, which might not interact with scavenger receptors, thereby evading Kupffer cell uptake. It may be a critical factor contributing to the slower clearance of the BAdV-3 vector from the host compared to the HAdV-5 vector, and allows the vector to persist for a longer duration in the liver and other organs [87]. To evaluate the in vitro safety of the BAdV-3 vector system, several cell lines were infected with BAdV-3, PAdV-3, or HAdV-5 vector and observed for an extended period for the state of the vector genome; only the linear episomal form of vector genomes was identified [12], suggesting that, similar to the HAdV-5 genome, the BAdV-3 genome does not integrate into the host genome.

Activation of innate mucosal immunity in mice by BAd-H5HA was investigated and compared with the HAd-H5HA group. Increased expression levels of TLR2, TLR3, TLR4, TLR7, and TLR9 were observed in the lungs of BAd-H5HA-inoculated animals shortly after i.n. inoculation [89]. There was a significant increase in the levels of interleukin (IL)-1α, IL-1β, IL-5, tumor necrosis factor (TNF)-α, leukemia inhibitory factor (LIF), IL-17, granulocyte colony-stimulating factor (G-CSF), C-C motif chemokine Ligand 4 (CCL4), C-C motif chemokine ligand 2 (CCL2), CXC motif chemokine ligand 2 (CXCL2), and granulocyte-macrophage colony-stimulating factor (GM-CSF) in the lungs of BAdV-3 vector-inoculated groups [89]. RNA-Seq analysis of the lung samples showed heightened differentially expressed (DE) genes of innate and adaptive immune response pathways, including TLR signaling, cytokine–cytokine receptor interaction, NOD-like receptor signaling, IL-17 signaling, and TNF signaling pathways [89]. In another study, increased expression of the aforementioned TLRs was observed in the liver and spleen post intravenous inoculation with BAd-GFP [87].

In a dose-escalation study comparing BAd-H5HA and HAd-H5HA expressing influenza H5N1 HA, mice were immunized i.n. or intramuscularly with either vector at a dose ranging from 1 ×10^6^ to 1 ×10^8^ plaque-forming units (PFU), followed by heterologous H5N1 influenza challenge. BAd-H5HA conferred complete protection at the 10^6^ PFU i.n. dose and 3 × 10^7^ PFU intramuscular dose. In comparison, HAd-H5HA achieved similar protection at the 3 × 10^7^ PFU i.n. dose, but even the highest dose, i.e., 1 × 10^8^ PFU, did not confer complete protection when administered intramuscularly. These results demonstrate that BAd-H5HA allows at least 30-fold dose sparing compared to the HAd-H5HA vector [10].

Although current data support the BAd-3 vector immunogenicity and efficacy, further preclinical evaluation is necessary to fully assess its safety profile. Studies in humanized murine models and nonhuman primates are essential to determine whether the vector elicits any undesirable immune activation, ensuring its suitability for use in human vaccination programs.

## 8. BAdV-3 Vector-Based Vaccines

BAdV-3 vectors have emerged as a promising platform for developing vaccines against a wide range of infectious diseases (Table 1). Initially, they were evaluated for their efficacy against multiple bovine respiratory viruses. Cotton rats immunized i.n., twice, 3 weeks apart, with 10^7^ PFU of replication-competent BAdV-3 vectors (BAV3.E3gD and BAV3.E3gDt) expressing either the full-length glycoprotein D (gD) or a truncated version of gD (gDt) of bovine herpes virus 1 (BoHV-1), respectively, elicited robust gD-specific IgG and IgA antibodies. BAV3.E3gDt induced greater gD-specific serum IgG titer (~4 log_10_) than BAV3.E3Gd. Mucosal IgA titers were similar, but BAV3.E3gD elicited a greater number of gD-specific IgA antibody-secreting cells (ASCs) in lungs than BAV3.E3gDt [73,90]. Subsequently, the effectiveness of the BAdV-3 vector-based BoHV-1 vaccine was evaluated in calves with significant BAdV-3-specific preexisting immunity. Three- to four-month-old calves were vaccinated i.n., twice, four weeks apart with 10^8^ PFU of BAV3.E3gD or BAV3.E3gDt. Both vectors induced a similar serum IgG titer (~10 log_10_) and mucosal IgA titer (~3 log_10_), conferring protection against challenge with 10^7^ PFU of BoHV-1 strain 108, two weeks post-boost [91]. These results suggest that i.n.-administered vaccines could circumvent BAdV-3-specific preexisting immunity and elicit protective immunity.

Similarly, a BAdV-3 vector (BAV360) was engineered to express the G protein of the bovine respiratory syncytial virus (BRSV), and another vector (BAV851) was designed to express two antigens, i.e., the G protein of BRSV along with the gDt glycoprotein of BoHV-1 [92]. The i.n. immunization of cotton rats with two doses of BAV360 or BAV851 at 10^7^ PFU, three weeks apart, resulted in robust antigen-specific systemic and mucosal immune responses. BAV851 elicited significant serum IgG and mucosal IgA titers against both antigens, i.e., gG and gD, but greater levels were generated against gD. Additionally, the VNT titer generated against BoHV-1 also exceeded that against BRSV. Replication-competent BAdV-3 vectors (BAV331 and BAV338) expressing E2 glycoprotein of bovine viral diarrhea virus (BVDV) administered at 10^7^ PFU, i.n. to cotton rats, twice, 3 weeks apart, elicited E2-specific systemic IgG and IgA antibodies in the nasal secretions and lung washes. The BAV338 vector elicited significantly greater levels of serum IgG and IgA ASC than the BAV331 vector [93]. These findings imply that replication-competent BAdV-3 vectors hold promise for developing effective vaccines for cattle viruses.

To design a mucosal vaccine targeting tuberculosis, replication-defective BAdV-3 (BAdv^85C5^) and HAdV-5 (HAdv^85C5^) vectors expressing the mycobacterial Ag85B-p25 epitope and the autophagy-inducing peptide C5 (AIP-C5) were developed [94]. Dendritic cells infected with BAdv^85C5^ exhibited an increased transcriptome associated with antigen processing and sorting endosomes to lysosomes compared to HAdv^85C5^-infected cells. BAdv^85C5^-infected dendritic cells demonstrated improved in vitro presentation of the Ag85B-p25 epitope to CD4+ T cells, with galectin (*Lgals*-3 and *Lgals*-8) and the autophagy pathway playing crucial roles [94]. Mice previously vaccinated with BCG (Bacillus Calmette-Guérin) were i.n. boosted with 10^7^ PFU of BAdv^85C5^ or HAdv^85C5^, followed by a challenge with 100 colony-forming units (CFU) of virulent aerosolized *Mycobacterium tuberculosis* (Mtb). BAdv^85C5^ vaccination resulted in an >1.4-log_10_ reduction in Mtb lung burden [94]. Meanwhile, single i.n. vaccination with BAdv^85C5^ led to an >0.5-log_10_ reduction in Mtb lung burden. This protection was linked to the significant expansion of CD8+ and CD4+ effector cells, lung-resident memory (CD103+/CD69+) cells, and central memory T cells [94]. The findings demonstrate that BAdv^85C5^ is a promising mucosal vaccine candidate for tuberculosis.

**Table 1:** BAdV-3 vector-based vaccines.

Vector Name	Deleted Region	Foreign Gene Cassette	Pathogen	Experimental Animal Used	Dose, Route, and Regimen	Study Outcome	References
BAV3.E3gD	E3	Full-length glycoprotein D (gD)	Bovine herpesvirus-1 (BoHV-1)	Cotton rats and calves	Cotton rats: 10^7^ PFU, i.n., twice, 3 weeks apart Calves: 10^8^ PFU, i.n., twice, 4 weeks apart	In cotton rats, BAV3.E3gDt induced greater gD-specific serum IgG titer (~4 log_10_) than BAV3.E3gD (2 log_10_). Mucosal IgA titers were similar (~2.5 log_10_); however, BAV3.E3gD elicited a greater number of gD-specific IgA antibody-secreting cells (ASCs) (30 ASC/million) in the lungs than BAV3.E3gDt (22 ASC/million). In calves, BAV3.E3gD and BAV3.E3gDt induced similar serum IgG titer (~10 log_10_) and mucosal IgA titer (~3 log_10_). Upon challenge with BoHV-1, calves immunized with either vaccine demonstrated lower clinical scores and less nasal viral shedding than the mock group.	[73,91]
BAV3.E3gDt	E3	Truncated glycoprotein D (gDt)	BoHV-1	Cotton rats and calves			
BAV331 and BAV338	E3	Glycoprotein E2 (gE2)	Bovine viral diarrhea virus	Cotton rats	10^7^ PFU, i.n., twice, 3 weeks apart	BAV338 vectors elicited significantly higher E2-specific systemic IgG titer (>1:256) than the BAV331 vector (>1:64). Both vectors elicited IgA antibodies in the nasal secretions and lung washes (≥1:16). BAV338 also elicited significantly greater IgA ASC in lungs (~10 ASC/million) than the BAV331 vector (~5 ASC/million).	[93]
BAV360	E3	Glycoprotein G (gG)	Bovine respiratory syncytial virus (BSRV)	Cotton rats	10^7^ PFU, i.n., twice, 3 weeks apart	BAV360 elicited a significant level of gG-specific IgG titer (2 log_10_) in serum, IgA titers (3 log_2_) in nasal secretions, and BRSV-specific VNT titers (6 log_2_). Further, BAV851 elicited serum IgG titer specific to gG (~3 log_10_) and gD (4 log_10_). Mucosal IgA titer was also elicited against gG (3 log_2_) and gD (5 log_2_). Additionally, BoHV-1-specific VNT titer (~7 log_2_) and BRSV-specific VNT titer (8 log_2_) were also induced.	[92]
BAV851	E3	gDt and gG	BoHV-1 and BSRV	Cotton rats			
BAd-H5HA	E1 and E3	Hemagglutinin (HA)	Influenza virus [A/Hong Kong/156/97(H5N1)]	BALB/c mice	10^6^ to 10^8^ PFU, i.n. or intramuscular, once	BAd-H5HA vector elicited high levels of anti-HA IgG_1_, IgG_2a_, and IgG_2b_ in serum and IgA antibodies in lung wash and nasal wash, along with interferon (IFN) γ-secreting HA_518–526_ peptide-specific CD8+ T cells in spleenocytes and lymph nodes. Mice were fully protected from heterologous H5N1 influenza challenge at the 10^6^ PFU i.n. dose and 3 × 10^7^ PFU intramuscular dose.	[10]
BAdv^85C5^	E1 and E3	Ag85B-p25 epitope and autophagy-inducing peptide C5 (AIP-C5)	*Mycobacterium tuberculosis*	C57BL/6 mice	10^7^ PFU, i.n.,	BCG vaccinated mice were i.n. boosted with BAdv^85C5^ and challenged with 100 colony-forming units (CFU) of virulent aerosolized *Mycobacterium tuberculosis* (Mtb). There was >1.4-log_10_ reduction in Mtb lung burden and ~0.8 log_10_ reduction in spleen Mtb load. Meanwhile, single i.n. vaccination with BAdv^85C5^ led to >0.5-log_10_ reduction in Mtb lung burden and ≥1 log_10_ reduction in spleen Mtb load.	[94]
BAd-FullHA-C5	E1 and E3	HA and AIP-C5	Influenza virus [A/Vietnam/1203/2004(H5N1)]	BALB/c mice	10^7^ to 3 × 10^7^ PFU, i.n.	The vectors with SP-M2e-HA2-Tri and SP-HAstem-C5 performed best across two studies and were further evaluated using HAdV vector prime and BAdV vector boost approach with the vector expressing FullHA-C5 as a positive control. SP-HAstem-C5 was the best candidate and provided complete protection following homologous (H5N1) [no mortality and ~3 log_10_ TCID_50_/mL reduction in lung viral load] or heterosubtypic (group 1, H1N1) [no mortality and ~1 log_10_ TCID_50_/mL reduction in lung viral load] virus challenge. Meanwhile, 80% protection and ~2 log_10_ TCID_50_/mL reduction in lung viral load were observed against group 2 (H3N2) virus challenge.	[78]
BAd-EP-HAstem-C5	E1 and E3	HA stem region, immunoglobulin excretory signal peptide (EP), and AIP-C5	Influenza virus [A/Vietnam/1203/2004(H5N1)]	BALB/c mice			
BAd-EP-HAstem-4M2e-C5	E1 and E3	HA stem region, EP, extracellular domains of matrix protein 2 (M2e), and AIP-C5	Influenza virus [A/Vietnam/1203/2004(H5N1) and A/Anhui/1YK_RG03/2013(H7N9)]	BALB/c mice			
BAd-SP-HAstem-C5	E1 and E3	HA stem region, HA signal peptide (SP), and AIP-C5	Influenza virus [A/Vietnam/1203/2004(H5N1)]	BALB/c mice			
BAd-SP-HAstem-4M2e-C5	E1 and E3	HA stem region, SP, M2e, and AIP-C5	Influenza virus [A/Vietnam/1203/2004(H5N1) and A/Anhui/1YK_RG03/2013(H7N9)]	BALB/c mice			

E1, early region 1; E3, early region 3; VNT, virus neutralization test; i.n., intranasal; PFU, plaque-forming units.

The BAdV-3 vector system has also been employed to develop vaccines against influenza viruses. BAdV-3 vector (BAd-H5HA) expressing influenza H5N1 HA, when inoculated i.n. or intramuscularly as a single dose in mice at varying doses ranging from 1 × 10^6^ to 1 × 10^8^ PFU, elicited high levels of anti-HA IgG_1_, IgG_2a_, IgG_2b_, and IgA antibodies, along with interferon (IFN) γ-secreting CD8+ T cells specific for the H-2K^d^-restricted HA_518–526_ epitope (IYSTVASSL). The vaccine performed better than the HAdV-5 vector expressing the same antigen and conferred complete protection against heterologous H5N1 influenza challenge, even at the lowest vaccine dose of 1 × 10^6^ PFU [10].

Continuous mutations in the influenza HA head domain lead to mismatches between vaccine and circulating field strains, making a broadly protective universal influenza vaccine imperative. Using conserved influenza virus proteins for vaccine development may pave the way out of this bottleneck [95]. BAdV-3 and HAdV-5 vector platforms expressing the HA stem region, HA2, trimerization motif (Tri), excretory peptide (EP), HA signal peptide (SP), and extracellular domains of matrix protein 2 (M2e) along with AIP-C5 were evaluated in immunogenicity and protection studies. For Study #1, mice were immunized i.n. with 3 × 10^7^ PFU of HAd-M2e-HA2, HAd-SP-M2e-HA2, and HAd-SP-M2e-HA2-Tri [78]. The HAd-SP-M2e-HA2-Tri vaccine performed best, eliciting high titers of HA-specific IgG, IgG_1_, and IgG_2a_, and IgA antibodies and INF-γ or IL-2 secreting HA2-specific T cells, leading to protection from homologous H5N1 influenza virus challenge. In Study #2, mice were primed i.n. with 10^7^ PFU of BAd-FullHA-C5, BAd-EP-HAstem-C5, BAd-EP-HAstem-4M2e-C5, BAd-SP-HAstem-C5, and BAd-SP-HAstem-4M2e-C5 and boosted with 10^8^ PFU of HAdV vectors matching the priming antigen 4 weeks later [78]. Apart from FullHA-C5 (positive control), SP-HAstem-C5 was the best performer and elicited high titers of HA-specific IgG, IgG_1_, and IgG_2a_ and IgA antibodies, along with INF-γ or IL-2 secreting HA2-specific T cells, and protected from homologous H5N1 influenza virus challenge.

The best candidates from the first two studies, i.e., SP-M2e-HA2-Tri and SP-HAstem-C5, were further evaluated in Study #3 using an HAdV vector prime (10^8^ PFU i.n.) and BAdV vector boost approach (3 × 10^7^ PFU i.n.). SP-HAstem-C5 was the best candidate and provided complete protection following homologous (H5N1) or heterosubtypic group 1 (H1N1) virus challenge, and 80% protection against group 2 (H3N2) virus challenge [78]. These results emphasize the potential of the BAdV vector platform, incorporating conserved influenza virus antigens, to develop a universal influenza vaccine.

To assess the effectiveness of the BAdV-3 vector in evading high levels of preexisting immunity to HAdV-5 vectors, naïve and HAdV-5-primed mice were subjected to immunization with BAd-H5HA [85]. Under conditions of significantly high preexisting immunity, mice vaccinated with BAd-H5HA demonstrated an approximately 2.8-fold increase in hemagglutination inhibition (HI) titers and a 2.3-fold rise in the percentage of HA-specific CD8+ T cells compared to levels observed in naïve mice administered HAd-H5HA. Despite the presence of high levels of HAdV-5-specific neutralizing antibody titers (2133 ± 660), mice immunized with BAd-H5HA did not exhibit any reductions in HA-specific humoral or CMI responses [85]. Immunization of both naïve and HAdV-primed mice with BAd-H5HA provided complete protection from morbidity and mortality after exposure to a potentially lethal H5N1 challenge (A/Hong Kong/483/97) [85]. In another study conducted in a mouse model for breast cancer, both HAdV-5 and BAdV-3 vectors demonstrated higher levels of transgene expression after intratumoral injections in the presence of heterologous vector immunity [96]. These findings indicate the lack of cross-reactivity between HAdV-5 and BAdV-3 and emphasize the use of a heterologous prime-boost approach involving the sequential administration of different AdV vectors to circumvent preexisting vector immunity.

## 9. Conclusions and Future Directions

BAdV vectors have emerged as a promising platform for developing vaccines against a wide range of infectious diseases. However, certain limitations need to be addressed. There is still limited data on the long-term safety of BAdV-3 vectors across humans and different animal species, and our understanding of the immune response generated against BAdV-3 vectors in different species is limited. Large-scale production of these vectors in an industrial setting poses another challenge, as there is no certified cell line. Another concern is the development of vector-specific immunity after priming vaccination, which could diminish its effectiveness upon repeated administration. 

As research continues to refine and expand the use of BAdV vectors, their role in the development of next-generation vaccines is expected to grow. Besides BAdV-3, other BAdVs need to be explored further to better understand their pathogenesis and for the development of novel vector systems. The potential of the BAdV-3 vector-based vaccine platform should be further investigated in different animal models, such as ferrets and nonhuman primates. Vectors containing further deletions in the E1, E3, and E4 regions can be generated to expand the foreign gene insertion capacity. In addition, a certified cell line that supports the replication of BAdV-based vectors should be developed. This cell line can rescue the recombinant vectors and grow the recombinants to high titers. Further studies are required to determine the best route of administration, optimum prime and boost dose, durability of immune responses, frequency of vector immunization, safety, and efficacy of the BAdV-based vector platform. A rare side effect that has been linked to HAdV and ChAdV vector-based vaccines and has been making the headlines lately is vaccine-induced immune thrombotic thrombocytopenia (VITT). This occurs due to the interaction of viral surface proteins with blood clotting factors, resulting in the formation of blood clots and reduction in platelet levels post-vaccination. It will be interesting to study whether BAdV-3 also interacts with the blood factors and causes VITT.

## References

[1] Damania B.A., Howley P.M., Knipe D.M., Cohen J.L. (2021). Adenoviridae: The Viruses and Their Replication. Fields Virology: DNA Viruses.

[2] MacLachlan N.J., Dubovi E.J. (2017). Adenoviridae. Fenner’s Veterinary Virology.

[3] Benkő M., Aoki K., Arnberg N., Davison A.J., Echavarría M., Hess M., Jones M.S., Kaján G.L., Kajon A.E., Mittal S.K. Family: Adenoviridae. https://ictv.global/report/chapter/adenoviridae/adenoviridae.

[4] Vidovszky M.Z., Böszörményi K.P., Surján A., Varga T., Dán Á., Benkő M., Harrach B. (2022). First DNA sequence proof for the occurrence of bovine adenovirus types 10 and 11 in continental Europe. Transbound. Emerg. Dis..

[5] Hong L., Li J., Zeng W., Li Y., Yu C., Zhao S., Chen L., Feng Y. (2025). The seroprevalence of adenoviruses since 2000(1). Emerg. Microbes Infect..

[6] Alhashimi M., Elkashif A., Sayedahmed E.E., Mittal S.K. (2021). Nonhuman Adenoviral Vector-Based Platforms and Their Utility in Designing Next Generation of Vaccines for Infectious Diseases. Viruses.

[7] Moffatt S., Hays J., HogenEsch H., Mittal S.K. (2000). Circumvention of vector-specific neutralizing antibody response by alternating use of human and non-human adenoviruses: Implications in gene therapy. Virology.

[8] Sharma A., Tandon M., Ahi Y.S., Bangari D.S., Vemulapalli R., Mittal S.K. (2010). Evaluation of cross-reactive cell-mediated immune responses among human, bovine and porcine adenoviruses. Gene Ther..

[9] Li X., Bangari D.S., Sharma A., Mittal S.K. (2009). Bovine adenovirus serotype 3 utilizes sialic acid as a cellular receptor for virus entry. Virology.

[10] Sayedahmed E.E., Hassan A.O., Kumari R., Cao W., Gangappa S., York I., Sambhara S., Mittal S.K. (2018). A Bovine Adenoviral Vector-Based H5N1 Influenza -Vaccine Provides Enhanced Immunogenicity and Protection at a Significantly Low Dose. Mol. Ther. Methods Clin. Dev..

[11] Sharma A., Bangari D.S., Tandon M., Pandey A., HogenEsch H., Mittal S.K. (2009). Comparative analysis of vector biodistribution, persistence and gene expression following intravenous delivery of bovine, porcine and human adenoviral vectors in a mouse model. Virology.

[12] Sharma A., Bangari D.S., Vemula S.V., Mittal S.K. (2011). Persistence and the state of bovine and porcine adenoviral vector genomes in human and nonhuman cell lines. Virus Res..

[13] Fent G.M., Fulton R.W., Saliki J.T., Caseltine S.L., Lehmkuhl H.D., Confer A.W., Purdy C.W., Briggs R.E., Loan R.W., Duff G.C. (2002). Bovine adenovirus serotype 7 infections in postweaning calves. Am. J. Vet. Res..

[14] Graham D.A., Calvert V., Benkö M., Curran W., Wylie M., Snodden D.A., Moffet D.A., Papp T., Adair B.M., Smyth J.A. (2005). Isolation of bovine adenovirus serotype 6 from a calf in the United Kingdom. Vet. Rec..

[15] Lehmkuhl H.D., Briggs R.E., Cutlip R.C. (1998). Survey for antibodies to bovine adenoviruses in six- to nine-month-old feedyard cattle. Am. J. Vet. Res..

[16] Thompson K.G., Thomson G.W., Henry J.N. (1981). Alimentary tract manifestations of bovine adenovirus infections. Can. Vet. J..

[17] Darbyshire J.H., Dawson P.S., Lamont P.H., Ostler D.C., Pereira H.G. (1965). A new adenovirus serotype of bovine origin. J. Comp. Pathol..

[18] Klein M., Earley E., Zellat J. (1959). Isolation from cattle of a virus related to human adenovirus. Proc. Soc. Exp. Biol. Med..

[19] Klein M., Zellat J., Michaelson T.C. (1960). A new bovine adenovirus related to human adenovirus. Proc. Soc. Exp. Biol. Med..

[20] Bürki F. (1990). Bovine Adenoviruses. Virus Infections of Ruminants.

[21] Fulton R.W., Anderson D.E., Rings D.M. (2009). Viral Diseases of the Bovine Respiratory Tract. Food Animal Practice.

[22] Aldasy P., Csontos L., Bartha A. (1965). Pneumo-enteritis in calves caused by Adenoviruses. Acta Vet. Acad. Sci. Hung..

[23] Darbyshire J.H., Jennings A.R., Omar A.R., Dawson P.S., Lamont P.H. (1965). Association of adenoviruses with bovine respiratory diseases. Nature.

[24] Coria M.F., McClurkin A.W., Cutlip R.C., Ritchie A.E. (1975). Isolation and characterization of bovine adenovirus type 5 associated with “weak calf syndrome”. Arch. Virol..

[25] Wilcox G.E. (1969). Isolation of adenoviruses from cattle with conjunctivitis and kerato-conjunctivitis. Aust. Vet. J..

[26] Bartha A., Máthé S., Aldásy P. (1970). New serotype 8 of bovine adenoviruses. Acta Vet. Acad. Sci. Hung..

[27] Darbyshire J.H., Kinch D.A., Jennings A.R. (1969). Experimental infection of calves with bovine adenovirus types 1 and 2. Res. Vet. Sci..

[28] Saxegaard F., Bratberg B. (1971). Isolation of bovine adenovirus type 1 from a calf with pneumo-enteritis. Acta Vet. Scand..

[29] Mittal S.K., Tikoo S.K., Van Donkersgoed J., Beskorwayne T., Godson D.L., Babiuk L.A. (1999). Experimental inoculation of heifers with bovine adenovirus type 3. Can. J. Vet. Res..

[30] Mittal S.K., Middleton D.M., Tikoo S.K., Babiuk L.A. (1995). Pathogenesis and immunogenicity of bovine adenovirus type 3 in cotton rats (Sigmodon hispidus). Virology.

[31] Jesse S.T., Ciurkiewicz M., Siesenop U., Spitzbarth I., Osterhaus A., Baumgärtner W., Ludlow M. (2022). Molecular characterization of a bovine adenovirus type 7 (Bovine Atadenovirus F) strain isolated from a systemically infected calf in Germany. Virol. J..

[32] Stalber E., Renshaw H.W., Boro C., Mattson D., Frank F.W. (1976). Isolation of a subgroup two adenovirus from calf with weak calf syndrome. Can. J. Comp. Med..

[33] Yokoi K., Okazaki H., Inahara K., Hatama S. (2009). Prevalence of eight bovine viruses in sika deer *(Cervus nippon yesoensis*) in Japan. Vet. Rec..

[34] Adair B.M., McKillop E.R., Smyth J.A., Curran W.L., McNulty M.S. (1996). Bovine adenovirus type 10: Properties of viruses isolated from cases of bovine haemorrhagic enterocolitis. Vet. Rec..

[35] Smyth J.A., Benkö M., Moffett D.A., Harrach B. (1996). Bovine adenovirus type 10 identified in fatal cases of adenovirus-associated enteric disease in cattle by in situ hybridization. J. Clin. Microbiol..

[36] Darbyshire J.H. (1966). Oncogenicity of bovine adenovirus type 3 in hamsters. Nature.

[37] Rondhuis P.R. (1973). Induction of tumors in hamsters with a bovine adenovirus strain (serotype 8). Arch. Die Gesamte Virusforsch..

[38] Reddy P.S., Idamakanti N., Zakhartchouk A.N., Baxi M.K., Lee J.B., Pyne C., Babiuk L.A., Tikoo S.K. (1998). Nucleotide sequence, genome organization, and transcription map of bovine adenovirus type 3. J. Virol..

[39] Mittal S.K., Prevec L., Babiuk L.A., Graham F.L. (1993). Sequence analysis of bovine adenovirus type 3 early region 3 and fibre protein genes. J. Gen. Virol..

[40] Ruigrok R.W., Barge A., Mittal S.K., Jacrot B. (1994). The fibre of bovine adenovirus type 3 is very long but bent. J. Gen. Virol..

[41] Nguyen T.H., Vidovszky M.Z., Ballmann M.Z., Sanz-Gaitero M., Singh A.K., Harrach B., Benkő M., van Raaij M.J. (2015). Crystal structure of the fibre head domain of bovine adenovirus 4, a ruminant atadenovirus. Virol. J..

[42] Wickham T.J., Mathias P., Cheresh D.A., Nemerow G.R. (1993). Integrins alpha v beta 3 and alpha v beta 5 promote adenovirus internalization but not virus attachment. Cell.

[43] Bergelson J.M., Cunningham J.A., Droguett G., Kurt-Jones E.A., Krithivas A., Hong J.S., Horwitz M.S., Crowell R.L., Finberg R.W. (1997). Isolation of a common receptor for Coxsackie B viruses and adenoviruses 2 and 5. Science.

[44] Bangari D.S., Sharma A., Mittal S.K. (2005). Bovine adenovirus type 3 internalization is independent of primary receptors of human adenovirus type 5 and porcine adenovirus type 3. Biochem. Biophys. Res. Commun..

[45] Snijder J., Reddy V.S., May E.R., Roos W.H., Nemerow G.R., Wuite G.J. (2013). Integrin and defensin modulate the mechanical properties of adenovirus. J. Virol..

[46] Wiethoff C.M., Wodrich H., Gerace L., Nemerow G.R. (2005). Adenovirus protein VI mediates membrane disruption following capsid disassembly. J. Virol..

[47] Bremner K.H., Scherer J., Yi J., Vershinin M., Gross S.P., Vallee R.B. (2009). Adenovirus transport via direct interaction of cytoplasmic dynein with the viral capsid hexon subunit. Cell Host Microbe.

[48] Cassany A., Ragues J., Guan T., Bégu D., Wodrich H., Kann M., Nemerow G.R., Gerace L. (2015). Nuclear import of adenovirus DNA involves direct interaction of hexon with an N-terminal domain of the nucleoporin Nup214. J. Virol..

[49] Strunze S., Engelke M.F., Wang I.H., Puntener D., Boucke K., Schleich S., Way M., Schoenenberger P., Burckhardt C.J., Greber U.F. (2011). Kinesin-1-mediated capsid disassembly and disruption of the nuclear pore complex promote virus infection. Cell Host Microbe.

[50] Trotman L.C., Mosberger N., Fornerod M., Stidwill R.P., Greber U.F. (2001). Import of adenovirus DNA involves the nuclear pore complex receptor CAN/Nup214 and histone H1. Nat. Cell Biol..

[51] Reddy P.S., Chen Y., Idamakanti N., Pyne C., Babiuk L.A., Tikoo S.K. (1999). Characterization of early region 1 and pIX of bovine adenovirus-3. Virology.

[52] White E. (2001). Regulation of the cell cycle and apoptosis by the oncogenes of adenovirus. Oncogene.

[53] Frisch S.M., Mymryk J.S. (2002). Adenovirus-5 E1A: Paradox and paradigm. Nat. Rev. Mol. Cell Biol..

[54] Zhou Y., Reddy P.S., Babiuk L.A., Tikoo S.K. (2001). Bovine adenovirus type 3 E1B(small) protein is essential for growth in bovine fibroblast cells. Virology.

[55] Mul Y.M., Verrijzer C.P., van der Vliet P.C. (1990). Transcription factors NFI and NFIII/oct-1 function independently, employing different mechanisms to enhance adenovirus DNA replication. J. Virol..

[56] Hoeben R.C., Uil T.G. (2013). Adenovirus DNA replication. Cold Spring Harb. Perspect. Biol..

[57] Webster A., Leith I.R., Nicholson J., Hounsell J., Hay R.T. (1997). Role of preterminal protein processing in adenovirus replication. J. Virol..

[58] de Jong R.N., van der Vliet P.C., Brenkman A.B. (2003). Adenovirus DNA replication: Protein priming, jumping back and the role of the DNA binding protein DBP. Curr. Top. Microbiol. Immunol..

[59] van Amerongen H., van Grondelle R., van der Vliet P.C. (1987). Interaction between adenovirus DNA-binding protein and single-stranded polynucleotides studied by circular dichroism and ultraviolet absorption. Biochemistry.

[60] Charman M., Herrmann C., Weitzman M.D. (2019). Viral and cellular interactions during adenovirus DNA replication. FEBS Lett..

[61] Graham F.L. (1984). Covalently closed circles of human adenovirus DNA are infectious. EMBO J..

[62] Russell W.C. (2009). Adenoviruses: Update on structure and function. J. Gen. Virol..

[63] Said A., Wang W., Woldermariam T., Tikoo S.K. (2018). Domains of bovine adenovirus-3 protein 22K involved in interacting with viral protein 52K and cellular importins α-5/α-7. Virology.

[64] Hong S.S., Szolajska E., Schoehn G., Franqueville L., Myhre S., Lindholm L., Ruigrok R.W., Boulanger P., Chroboczek J. (2005). The 100K-chaperone protein from adenovirus serotype 2 (Subgroup C) assists in trimerization and nuclear localization of hexons from subgroups C and B adenoviruses. J. Mol. Biol..

[65] Ostapchuk P., Hearing P. (2005). Control of adenovirus packaging. J. Cell. Biochem..

[66] Ahi Y.S., Vemula S.V., Hassan A.O., Costakes G., Stauffacher C., Mittal S.K. (2015). Adenoviral L4 33K forms ring-like oligomers and stimulates ATPase activity of IVa2: Implications in viral genome packaging. Front. Microbiol..

[67] Ahi Y.S., Mittal S.K. (2016). Components of Adenovirus Genome Packaging. Front. Microbiol..

[68] Ahi Y.S., Hassan A.O., Vemula S.V., Li K., Jiang W., Zhang G.J., Mittal S.K. (2017). Adenoviral E4 34K protein interacts with virus packaging components and may serve as the putative portal. Sci. Rep..

[69] Gupta S.P., Shaik B., Prabhakar Y.S., Gupta S.P. (2017). Advances in Studies on Adenovirus Proteases and Their Inhibitors. Viral Proteases and Their Inhibitors.

[70] Zhang H., Wang H., An Y., Chen Z. (2023). Construction and application of adenoviral vectors. Mol. Ther. Nucleic Acids.

[71] Bangari D.S., Shukla S., Mittal S.K. (2005). Comparative transduction efficiencies of human and nonhuman adenoviral vectors in human, murine, bovine, and porcine cells in culture. Biochem. Biophys. Res. Commun..

[72] Mittal S.K., Prevec L., Graham F.L., Babiuk L.A. (1995). Development of a bovine adenovirus type 3-based expression vector. J. Gen. Virol..

[73] Zakhartchouk A.N., Reddy P.S., Baxi M., Baca-Estrada M.E., Mehtali M., Babiuk L.A., Tikoo S.K. (1998). Construction and characterization of E3-deleted bovine adenovirus type 3 expressing full-length and truncated form of bovine herpesvirus type 1 glycoprotein gD. Virology.

[74] Mittal S.K., Middleton D.M., Tikoo S.K., Prevec L., Graham F.L., Babiuk L.A. (1996). Pathology and immunogenicity in the cotton rat (Sigmodon hispidus) model after infection with a bovine adenovirus type 3 recombinant virus expressing the firefly luciferase gene. J. Gen. Virol..

[75] van Olphen A.L., Mittal S.K. (1999). Generation of infectious genome of bovine adenovirus type 3 by homologous recombination in bacteria. J. Virol. Methods.

[76] Reddy P.S., Idamakanti N., Chen Y., Whale T., Babiuk L.A., Mehtali M., Tikoo S.K. (1999). Replication-defective bovine adenovirus type 3 as an expression vector. J. Virol..

[77] Du E., Tikoo S.K. (2010). Efficient replication and generation of recombinant bovine adenovirus-3 in nonbovine cotton rat lung cells expressing I-SceI endonuclease. J. Gene Med..

[78] Wang W.C., Sayedahmed E.E., Alhashimi M., Elkashif A., Gairola V., Murala M.S.T., Sambhara S., Mittal S.K. (2025). Adenoviral Vector-Based Vaccine Expressing Hemagglutinin Stem Region with Autophagy-Inducing Peptide Confers Cross-Protection Against Group 1 and 2 Influenza A Viruses. Vaccines.

[79] Ayalew L.E., Kumar P., Gaba A., Makadiya N., Tikoo S.K. (2015). Bovine adenovirus-3 as a vaccine delivery vehicle. Vaccine.

[80] Baxi M.K., Robertson J., Babiuk L.A., Tikoo S.K. (2001). Mutational analysis of early region 4 of bovine adenovirus type 3. Virology.

[81] van Olphen A.L., Tikoo S.K., Mittal S.K. (2002). Characterization of bovine adenovirus type 3 E1 proteins and isolation of E1-expressing cell lines. Virology.

[82] Patel A.K., Tikoo S.K. (2006). 293T cells expressing simian virus 40 T antigen are semi-permissive to bovine adenovirus type 3 infection. J. Gen. Virol..

[83] Zheng B., Mittal S.K., Graham F.L., Prevec L. (1994). The E1 sequence of bovine adenovirus type 3 and complementation of human adenovirus type 5 E1A function in bovine cells. Virus Res..

[84] van Olphen A.L., Mittal S.K. (2002). Development and characterization of bovine x human hybrid cell lines that efficiently support the replication of both wild-type bovine and human adenoviruses and those with E1 deleted. J. Virol..

[85] Singh N., Pandey A., Jayashankar L., Mittal S.K. (2008). Bovine adenoviral vector-based H5N1 influenza vaccine overcomes exceptionally high levels of pre-existing immunity against human adenovirus. Mol. Ther..

[86] Kuchipudi S.V., Nelli R.K., Gontu A., Satyakumar R., Surendran Nair M., Subbiah M. (2021). Sialic Acid Receptors: The Key to Solving the Enigma of Zoonotic Virus Spillover. Viruses.

[87] Sharma A., Bangari D.S., Tandon M., Hogenesch H., Mittal S.K. (2010). Evaluation of innate immunity and vector toxicity following inoculation of bovine, porcine or human adenoviral vectors in a mouse model. Virus Res..

[88] Mittal S.K., Ahi Y.S., Vemula S.V., Curiel D.T. (2016). Xenogenic Adenoviral Vectors. Adenoviral Vectors for Gene Therapy.

[89] Sayedahmed E.E., Elshafie N.O., Zhang G., Mohammed S.I., Sambhara S., Mittal S.K. (2023). Enhancement of mucosal innate and adaptive immunity following intranasal immunization of mice with a bovine adenoviral vector. Front. Immunol..

[90] Zhang L., Gomis S., Tikoo S.K. (2005). Evaluation of promoters for foreign gene expression in the E3 region of bovine adenovirus type-3. Virus Res..

[91] Zakhartchouk A.N., Pyne C., Mutwiri G.K., Papp Z., Baca-Estrada M.E., Griebel P., Babiuk L.A., Tikoo S.K. (1999). Mucosal immunization of calves with recombinant bovine adenovirus-3: Induction of protective immunity to bovine herpesvirus-1. J. Gen. Virol..

[92] Brownlie R., Kumar P., Babiuk L.A., Tikoo S.K. (2015). Recombinant bovine adenovirus-3 co-expressing bovine respiratory syncytial virus glycoprotein G and truncated glycoprotein gD of bovine herpesvirus-1 induce immune responses in cotton rats. Mol. Biotechnol..

[93] Baxi M.K., Deregt D., Robertson J., Babiuk L.A., Schlapp T., Tikoo S.K. (2000). Recombinant bovine adenovirus type 3 expressing bovine viral diarrhea virus glycoprotein E2 induces an immune response in cotton rats. Virology.

[94] Khan A., Sayedahmed E.E., Singh V.K., Mishra A., Dorta-Estremera S., Nookala S., Canaday D.H., Chen M., Wang J., Sastry K.J. (2021). A recombinant bovine adenoviral mucosal vaccine expressing mycobacterial antigen-85B generates robust protection against tuberculosis in mice. Cell Rep. Med..

[95] Wang W.C., Sayedahmed E.E., Sambhara S., Mittal S.K. (2022). Progress towards the Development of a Universal Influenza Vaccine. Viruses.

[96] Tandon M., Sharma A., Vemula S.V., Bangari D.S., Mittal S.K. (2012). Sequential administration of bovine and human adenovirus vectors to overcome vector immunity in an immunocompetent mouse model of breast cancer. Virus Res..

